# Global γH2AX phosphorylation in *Drosophila* is reversed by the phosphatase Mts

**DOI:** 10.17912/micropub.biology.001474

**Published:** 2025-02-19

**Authors:** Zivkos Apostolou, Silke Krause, Peter B. Becker

**Affiliations:** 1 Biomedical Center, Molecular Biology Division, Ludwig-Maximilians-Universität München, Munich, Germany

## Abstract

The phosphorylation of the histone variant H2AX to form γH2AX is an early and critical histone modification during the DNA damage response. This phosphorylation has proven to be a highly specific molecular marker for tracking the initiation and resolution of DNA damage. In this study, we investigate the roles of three phosphatases in removing the ‘γ' phospho-epitope from H2AX in *Drosophila* Kc167 cells. We found that the bulk of the X-ray-induced γH2AX signal is erased by the PP2A-type phosphatase MTS (microtubule star).

**Figure 1. Global γH2AX phosphorylation in Drosophila is reversed by the phosphatase Mts:**
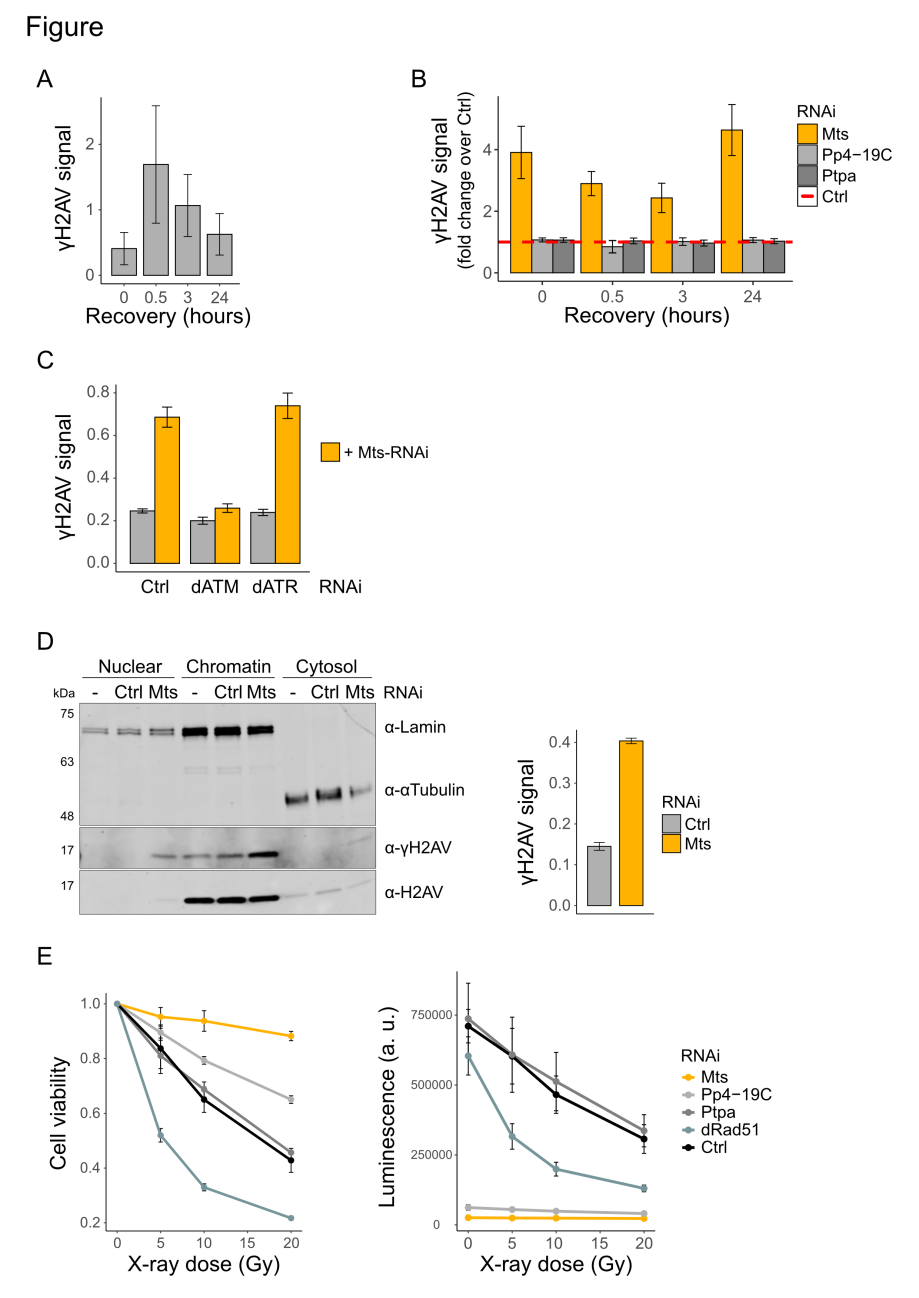
**A** ) Kinetics of γH2AV staining upon 10 Gy of X-ray irradiation Kc167 cells treated with an RNA interference (RNAi) against the irrelevant GST sequence. Samples at recovery time point "0" were not irradiated. γH2AV in whole-cell extracts were quantified on immunoblots. γH2AV signals were normalized to the corresponding H2AV signal. The error bars indicate standard error of the mean (SEM) of 4 biological replicates. **B** ) Kinetics of γH2AV removal upon X-ray irradiation in Kc167 cells, in which three different phosphatases have been depleted by RNAi. Cells were irradiated with 10 Gy of X-rays and collected at the indicated times. Samples at recovery time point "0" were not irradiated. γH2AV signals in whole-cell extracts were quantified on immunoblots. γH2AV signals were normalized to the corresponding H2AV signal. Plot shows the fold change of the γH2AV signal over the control sample [see (A)]. The error bars indicate standard error of the mean (SEM) of 3 biological replicates for the Mts samples or 4 for the other two phosphatases. **C** ) Increased γH2AV levels in *Mts* -depleted cells is ATM-dependent. Cells were depleted of dATM or dATR (along with a GST control) and then additionally treated with RNAi against *Mts* , as indicated. γH2AV signals in whole-cell extracts were quantified on immunoblots and normalized to the corresponding H2AV signal. Error bars indicate standard error of the mean (SEM) of 4 biological replicates. **D** ) Left: Persistent γH2AV is chromatin-bound in *Mts* -depleted cells. Control cells (with or without RNAi) and *Mts* -depleted cells were subjected to subcellular fractionation. Immunoblots were performed on nuclear, chromatin, and cytosolic fractions. Right: Quantification of the γH2AV signals from the chromatin fractions (left) by immunoblotting. γH2AV signals were normalized to the corresponding H2AV signals. Error bars indicate standard error of the mean (SEM) of 3 biological replicates. **E** ) Proliferation defects in *Mts* -depleted cells mask the effects of X-ray irradiation. Left: Cells in which the indicated factors were depleted were irradiated using the indicated dose of X-ray on day 4. The fraction of viable cells relative to non-irradiated conditions was measured using a luminescence-based assay (ATP-Glo) on day 7. Right: The raw intensity values of the samples in the right panel reveal that effect of RNAi on cell proliferation. Error bars indicate standard error of the mean (SEM) of 3 biological replicates.

## Description

The phosphorylation of the histone variant H2AX by ATM or ATR kinases serves as an evolutionary conserved mediator of DNA damage signaling (Kinner *et al* , 2008). C-terminally phosphorylated H2AX (γH2AX) is recognized by proteins involved in signal transduction and DNA repair complex assembly (Downs *et al* , 2004; Kleiner *et al* , 2015; Stucki *et al* , 2005). During the phases of recovery from DNA damage or adaptation, the damage signaling has to be cancelled by removal of the ‘γ' phospho-epitope. In yeast and mammals this is achieved by a range of different phosphatases (Ramos *et al* , 2019). Whereas in yeast this dephosphorylation may happen after removal of the H2A-H2B histone dimer from chromatin (Keogh *et al* , 2006), in mammals the chromatin-bound γH2AX is directly dephosphorylated (Chowdhury *et al* , 2005; Chowdhury *et al* , 2008; Nakada *et al* , 2008).

*Drosophila melanogaster* does not have a dedicated H2AX, but instead, the histone variant H2AV which otherwise looks like H2AZ,carries a C-terminal ‘γ' epitope that is phosphorylated by checkpoint kinases. Thus, H2AV combines the two functions of H2AZ in active promoter definition and H2AX as a mediator of DNA damage signaling (Baldi & Becker, 2013) .

It has been suggested that in *Drosophila* , the cancellation of γH2AX signaling does not involve phosphatases, but rather the exchange of γH2AV for unmodified H2AV by the DOMINO nucleosome remodeling complex, which is related to the mammalian SRCAP and P400 complexes (Kusch *et al* , 2004). The large DOMINO complex combines ATP-dependent histone variant exchange activity with histone acetyltransferase (HAT) activity, contributed by the HAT TIP60. Kusch et al., suggested that acetylation of γH2AV by TIP60 serves as a trigger for the exchange of γH2AV for an unmodified histone during the recovery from DNA damage repair.

We previously found that the *domino* gene gives rise to two alternative ATPase splice variants that define two distinct epigenetic regulators (Börner & Becker, 2016; Scacchetti *et al* , 2020). The short isoform, DOM-B, related to mammalian SRCAP, is mainly involved in incorporating the H2AV variant into the fly genome. The longer isoform, DOM-A, defines a remodeler related to mammalian EP400, as it also contains a TIP60 HAT module. DOM-A lacks the ARP6 subunit which is crucial for H2AV exchange (Scacchetti & Becker, 2021) . Accordingly, depletion of DOM-A does not affect bulk H2AV levels or distribution (Scacchetti *et al.* , 2020).

Since the DOM-A complex does not combine HAT and histone exchange activities as proposed (Kusch *et al.* , 2004), we revisited the involvement of the major *Drosophila* phosphatases in the reversion of the γH2AV signal. Thus, we considered the PP2A-type phosphatase MTS (microtubule star) (Snaith *et al* , 1996), the PP4-19C (Helps *et al* , 1998) and, as a control, the tyrosyl phosphatase PTPA (Hoof *et al* , 1998).

To investigate a possible role for the phosphatases in regulating γH2AV, we depleted the phosphatases MTS, PP4-19C and PTPA by RNA interference in Kc167 cells. Immunoblotting of whole cell extracts showed that exposure of these cells to 10 Gy of X-ray irradiation strongly induced the γH2AV signal after 30 minutes. The signal was reversed during 24 hours of recovery ( **Figure A** ). Depletion of *Mts* resulted in an increase of γH2AV levels compared to control cells at all times ( **Figure B).** In contrast, the γH2AV levels were not affected in cells depleted of *Pp4-19C* or *Ptpa* . Interestingly, *Mts* -depleted cells showed a significant increase of basal γH2AV in the absence of irradiation ( **Figure B** ), suggesting that MTS is continuously involved in signal cancellation in the context of steady-state DNA repair. In support of this conclusion, recombinant MTS was able to efficiently remove the ‘γ' phospho-epitope from H2AV, which had been acid-extracted from X-irradiated Kc167 cells.

We next asked whether the increased basal γH2AV levels in *Mts* -depleted cells is mediated by the kinases dATM (Tefu) or dATR (Mei-41). *Mts* -depletion was combined with RNA interference against *atm* or *mei-41* . Upon depletion of *dATM,* but not *dATR* the MTS-dependent elevation of γH2AV was counteracted, suggesting that ATM is majorly responsible for γ-phosphorylation upon X irradiation ( **Figure C** ).

Are the histones that carry the ‘γ' phospho-epitope in the *Mts* -depleted cells chromatin-bound? Biochemical subcellular fractionation of *Mts* -depleted cells shows a significant enrichment of γH2AV in the chromatin fraction over the control ( **Figure D** ), suggesting that the majority of γH2AV dephosphorylation occurs on chromatin-bound histones.

To investigate the sensitivity of *Mts* -depleted cells to irradiation, we assayed cell viability using an ATP-Glo luciferase assay after exposure to different levels of X-ray. Three days after irradiation, *Mts* -depleted cells exhibit the highest scores of viable cells compared to *Pp4-19C-* and *Ptpa* -depleted cells or control cells, contrary to expectations. As a control, the depletion of dRAD51 (spn-A), a protein involved in the repair of DNA breaks by homologous recombination, rendered cells particularly vulnerable to irradiation ( **Figure E, left** ). At first sight, this finding contrasts with the anticipated radiation-sensitive phenotype of a factor involved in DNA damage signaling. However, *Mts* -depleted cells essentially stop dividing, presumably driven by checkpoint responses ( **Figure E, right** ). This finding highlights the essential role of MTS in regulating cell division (Snaith *et al.* , 1996).

In conclusion, our findings demonstrate that *Mts* -depletion results in elevated γH2AV levels both under X-irradiation and non-irradiated conditions. The latter may be due to endogenous or programmed DNA damage. In this respect, MTS might function analogously to yeast PPH3 and human PP4 C (Chowdhury *et al.* , 2008; Keogh *et al.* , 2006). Furthermore, the persistence of γH2AV in chromatin of MTS-deficient cells highlights the critical role of MTS in globally removing the ‘γ' phospho-epitope from H2AV. Consistent with this, the *Drosophila* protein Twins (tws), a subunit of the heterotrimeric PP2A-type phosphatase, colocalizes with γH2AV and its depletion similarly increases γH2AV levels (Merigliano *et al* , 2017). While it remains possible that DOM-A exchanges γH2AV for unmodified H2AV under specific conditions and at certain genomic loci, recent findings suggest that the mammalian P400 complex is incapable of hydrolyzing ATP (Park *et al* , 2024). This observation supports the idea that the DOM-A complex might primarily function as a scaffold for the TIP60 HAT.

## Methods

**Cell culture:** *Drosophila* embryonic Kc167 cells (DGRC) were grown at 26°C in Schneider's *Drosophila* Medium (Thermo-Fischer, Cat. No. 21720024) supplemented with 10% FBS (Capricorn, Cat. No. FBS-12A) and 1% Penicillin-Streptomycin solution (Sigma-Aldrich, Cat. No. P-4333).

**RNA interference** was performed using dsRNA against the target sequence, which was generated by *in-vitro* transcription using the HiScribe T7 High Yield RNA Synthesis Kit (NEB, Cat. No. E2040S). In short, 6 μg of dsRNA was applied to 0.8x10 ^6^ cells in 0.5 ml serum-free medium in 12-well cell culture plates. After one hour of incubation at 26°C, 0.5 ml of medium supplemented with 20% FBS and 2% Penicillin-Streptomycin was added to reach final concentrations of 10% and 1% respectively. In Figure C, cells underwent two sequential rounds of RNAi. In the first round, cells were treated for 4 days with control dsRNA or dsRNA targeting dATM or dATR to establish the respective deficient backgrounds. This was followed by a second round of RNAi targeting control, dATM, dATR, or MTS. For the dsRNA control, we target the heterologous sequence of the glutathione-S-transferase (GST) gene of *Schistosoma japonicum* .

**X-ray irradiation:** Cells were irradiated with indicated dose (grays) in cell culture plates without the lid using a Faxitron CellRad X-ray source (130 kV, 5 mA).

**Cell fractionation** was performed as previously described (Méndez & Stillman, 2000) with the addition of 1x cOmplete EDTA-free Protease inhibitor (Roche, Cat. No. 5056489001) and 1x PhosStop (Roche, Cat. No. 04906845001) in Buffers A and B.

**Cell viability assay:** After three days of RNAi treatment, the cells were counted, and 10 ^6^ cells were re-treated with dsRNA as previously described. The cells were then diluted 1:3 in media, and 50 μl (approximately 1.5x10 ^4^ cells) were re-seeded into white 96-well microplates with clear bottoms (BERTHOLD Technologies, Cat. No. 24910). On the fourth day, cells were subjected to varying doses of X-irradiation and subsequently incubated at 26°C for an additional three days. Cell viability was then assessed using the CellTiter-Glo Luminescent Cell Viability Assay (Promega, Cat. No. G7571). Luminescence measurements were performed with a Tecan Infinite M1000 microplate reader.

## Reagents

**Table 1: Primer sequence for dsRNA synthesis**

**Table:** 

**Name**	**Primer sequence**
Mts_RNAi_F1	taatacgactcactatagggCACGAGGCGAGATTCCC
Mts_RNAi_R1	taatacgactcactatagggAAATGCCCGGTGACAGTG
Pp4-19C_RNAi_F1	taatacgactcactatagggTAGACCTGTGTGATTTGGCG
Pp4-19C_RNAi_R1	taatacgactcactatagggACGACTAACAACGCTGTCCC
Ptpa_RNAi_F1	taatacgactcactatagggGTAGTCGATCCTGGTGGCAT
Ptpa_RNAi_R1	taatacgactcactatagggCAGGTGGGCTGAGTGAATTT
Tefu(dATM)_RNAi_F1	taatacgactcactatagggGCTCATCCAAACTAGCGTAA
Tefu(dATM)_RNAi_R1	taatacgactcactatagggGCGTTCTGCTGGAAGATG
Mei-41(dATR)_RNAi_F2	taatacgactcactatagggGCTTGAAGGCATTTTCCTTAA
Mei-41(dATR)_RNAi_R2	taatacgactcactatagggAGAATACAAAGCACGTGGATA
Spn-A(dRad51)_RNAi_F2	taatacgactcactatagggGCACAATTAGCTCTCCCTGG
Spn-A(dRad51)_RNAi_R2	taatacgactcactatagggTTGAGACGGGATCCATTACC
GST(control)_F1	taaatacgactcactatagggAGAATGTCCCCTATACTAGGTTA
GST(control)_R1	taaatacgactcactatagggAGAACGCATCCAGGCACATTG

**Table 2: Antibodies for Immunoblots**

**Table:** 

**Name**	**Host Species**	**Dilution**	**Source**	**Reference**
H2AV	Rabbit (polyclonal)	1:1000	Laboratory of Peter B. Becker	(Börner & Becker, 2016)
γH2AV	Mouse (monoclonal)	1:1000	UNC93-5.2.1, Developmental Studies Hybridoma Bank	(Lake *et al* , 2013)
Lamin	Mouse (T40, monoclonal)	1:1000	Gift from Harald Saumweber	(Risau *et al* , 1981)
αTubulin	Mouse (monoclonal)	1:5000	Sigma-T9026	
